# Social Networks In Mid-To-Late-Life Adults And Cognitive Decline: The Framingham Heart Study

**DOI:** 10.1093/geroni/igaf122.4198

**Published:** 2025-12-31

**Authors:** Phillip Hwang

**Affiliations:** Boston University School of Public Health, Boston, Massachusetts, United States

## Abstract

Studies examining the relationship between social networks and cognitive decline mainly include older adults; less is known about the relationship when including adults earlier in life, such as middle age. We investigated the association between social network size among mid-to-late-life adults and cognitive decline in memory, language, and executive functioning. The study sample included 1,297 participants age 40-90 years from the Framingham Heart Study Offspring cohort. Social network size was determined by the Berkman-Syme Social Network Index (SNI), which was measured between 2005-2009, and categorized into four groups (e.g., low, low/medium, medium/high, and high). Cognitive factor scores for memory, language, and executive functioning were previously derived from neuropsychological tests. Linear mixed models were used to estimate mean annual change in each factor score associated with social network size, with the low SNI group as the reference. Models were adjusted for age, sex, education, body mass index, smoking, physical activity, and depressive symptoms. High SNI was significantly associated with declines in memory (β = -0.011; 95% CI = -0.02, -0.003) and executive functioning (β = -0.011; 95% CI = -0.02, -0.001). Medium/high SNI was also significantly associated with decline in memory (β = -0.013; 95% CI = -0.02, -0.005). Individuals with higher SNI, as compared to lower SNI, had greater proportions of prevalent dementia and incident dementia during the follow up period. These findings suggest that reverse causation impacts the association between social networks and cognitive decline and that seeking greater social support may be a sign of worsening cognitive function.

